# A New Method of Producing Local Anæsthesia of the Skin

**Published:** 1887-10

**Authors:** Henry J. Reynolds

**Affiliations:** Professor of Dermatology, College of Physicians and Surgeons, Chicago ; Professor of Skin and Genito-Urinary Diseases, Chicago Policlinic, etc., etc.


### A New Method of Producing Local Anæsthesia of the Skin.

Read in the Section on Medicine, Materia Medica, and Therapeutics, at the Thirty-Eighth Annual Meeting of the American Medical Association, June 7, 1887.

BY HENRY J. REYNOLDS, M.D.,

Professor of Dermatology, College of Physicians and Surgeons, Chicago ; Professor of Skin and Genito-Urinary Diseases, Chicago Policlinic, etc., etc.

Various means have been adopted for producing local anæsthesia of the skin, but heretofore none has been to any extent a success. Even cocaine, useful in most other tissues as a topical application, has absolutely no practical use as a local anæsthetic for the skin. Though local anæsthesia of the deeper parts may also be produced by the method which I am about to describe, I am only prepared to speak of its merits from a dermatological standpoint at present, but am nevertheless satisfied that the tissues can in this way be anæsthetized to a sufficient depth to render the method applicable in many cases of general surgery.

As cocaine is the anæsthetizing agent, the method therefore relates only to the manner in which the drug is applied and its absorption induced. Electricity is the means employed for this purpose. Knowing that solutions of cocaine were not readily absorbed by the skin, and therefore produce no anæsthetic effect, Dr. Wagner, of Vienna, about a year ago made experiments, the results of which he afterwards (*Wien Med. Blatt.*, 1886, vol. 9, page 161) presented to the Society of Physicians of Vienna in the form of a paper on that subject, with a view to ascertaining a more successful method of producing anæsthesia of the skin with this drug. He found that by saturating the positive electrode of a galvanic battery with a solution of cocaine, applying it to the skin and then applying the negative electrode a short distance from the positive, with a moderate current on, that successful anæsthesia could be produced. Since the publication of this article, though personally, as a rule, very skeptical regarding therapeutic innovations, I have been induced to make a number of experiments with the method, and to make frequent use of it in dermatological practice with quite a degree of satisfaction; and inasmuch therefore as I have in looking up the literature of the subject been unable to find any article touching upon the details or results of any personal experience with the method in this country, I feel justified in writing this article, in the hope that it may elicit discussion and the personal experience of others, and be the means of bringing to the more general notice of the profession of this country a procedure which I deem of much importance in medical and surgical practice. So far as I am aware, in fact, no article on the subject has appeared in medical literature other than the one just referred to. With this apology, and without going into details of my experiments, I trust that a brief description of the method and the results of my experience with it will be tolerated.

*Battery, strength of current, etc.*—I have used an eighteen cell McIntosh battery, but I think, in some cases, even a stronger current is necessary. For reasons not necessary to discuss at present the Faradic current will not answer; neither will the negative pole of the galvanic. The strength of the current must vary with the sensitiveness of the part, the size of the electrode, etc. It is always necessary to use the strongest current that can be borne, which may vary all the way from four or five cells (of the McIntosh battery) to twenty-four, but for which no rule can be laid down, the only guide being the feelings of the patient and the experience and discretion of the physician. Where the skin is very dense, the part not sensitive to electricity, and the electrode large owing to the greater surface necessary to be acted upon, (which thereby renders the current more diffused, and hence in a sense not so strong,) the current must be very strong, requiring perhaps twenty-four cells of the McIntosh instrument. The sponge on the electrode used for the solution should be of the finest and softest quality. When a strong current is used the irritation at the negative pole may be avoided by using a larger electrode.

*Strength of solution.*—In my experience the cocaine should not be used weaker than a five per cent. solution. It may vary, however, owing to circumstances, from a two to a twenty per cent. strength. Where the skin is thin and the part such as will tolerate a strong current, a two to five per cent. solution will answer. On the other hand, if the part be so sensitive that only a weak current can be employed, as in portions of the face, the solution to be effectual must not be less than a ten per cent. one.

*Mode of application.*—Saturate the positive electrode with the solution and place it directly upon the part to be anæsthetized. Place the negative electrode well saturated with water on some point near by. A more remote point, as the hand of the opposite side, for instance, will answer, but it will take longer time and a stronger solution to get the effect than when placed near by. In working about the face I think it is better to hold the negative electrode in the hand than to apply it to the face near the positive, as in this way a strong current will be better tolerated.

It is well for the operator to familiarize himself with the method by experiments upon his own person. The electrodes should be kept firmly pressed to the skin, the positive being now and then moistened as required with more of the solution, and the negative with more water. If the negative produces much irritation it may be occasionally shifted to another place.

*Time required to produce Anæsthesia.*—This depends of course upon the location, the strength of the solution, the strength of the current, etc. In the skin of the flexor surface of the forearm, if the negative pole be applied near by, a five per cent. solution with about ten cells, say, of a McIntosh battery in good order, will produce profound anæsthesia in five minutes. If the negative pole be placed in the hand of the opposite side about double that length of time will be required. Whenever the skin is dense a longer time, stronger solution, and stronger currents are necessary. In any case, the current should be allowed to run as described till anæsthesia is produced, as ascertained by gently pricking now and then with some sharp instrument.

*Duration of Anæsthesia.*—This of course varies more or less also with the circumstances; as a rule, however, the effect is rather transitory, lasting perhaps from five to fifteen minutes.

*Results in Practice.*—In the operation for the removal of superfluous hairs from the face by electrolysis, I have frequently used it. In this operation, inasmuch as the tissues still conduct the current, an ordinary electrical sensation is experienced by the patient, but the sharp, stinging, burning pain at the site of the needle, sometimes so acutely felt, is, when the method is judiciously employed, entirely done away with. In the removal of small warts, nævi, etc., from the face by the knife, it is even more satisfactory. One obstacle in the way of its successful employment in certain portions of the face is the extreme sensitiveness of the part, owing to which condition a sufficiently strong current cannot always be tolerated to induce the necessary absorption of the drug. In a case of felon of the palm at the junction of the middle finger, I made a very careful effort with a ten per cent. solution and sixteen cells of a McIntosh battery, to produce anæsthesia previous to lancing. In this case the method as I employed it was not a success. Failure in this case would be obvious for the following reasons: *First,* the skin was very thick and callous; *second,* owing to the extreme soreness not sufficient pressure with the electrode could be made to get the full benefit of the current; *third,* I think under the circumstances the current was not nearly as strong as it should have been. In dental surgery I think the method can also be used to advantage. I applied it in one case for Dr. W. H. Gale, of this city; the patient was a lady for whom two teeth were extracted. It is, of course, impossible to determine in such a case exactly how much benefit is derived from the procedure, but as nearly as could be ascertained, the pain in this case from the extraction was comparatively insignificant. Ten cells of the McIntosh battery were used with a ten per cent. solution.

From the foregoing it will be seen that while under favorable circumstances complete anæsthesia of the skin may be in this way produced, like every thing else, with our present knowledge of the method, it is not under all circumstances all that could be desired. Notwithstanding, it has unquestionably, sufficient merit to warrant its recommendation to the profession and its general adoption by those in dermatological practice especially, and I am satisfied that with further experience, more extended observation, improved appliances, and the more general adoption of the method, many of these apparent obstacles may be overcome.

In conclusion I may summarize as follows:

1. Complete anæsthesia of the skin may, under the proper conditions, be produced by cocaine applied in conjunction with the galvanic current.

2. In dermatological practice, the method, which, for want of a better name, I may term "cocaino-galvanism, galvano-cocainism, or galvano-cocaine anæsthesia" is the best means for the purpose at our command.

3. In dermatological practice it is preferable to the hypodermic injection of the drug inasmuch as it is painless and frequently more effectual.

4. The cocaine should not be used in less than a five per cent. solution.

5. The current should be as strong as the idiosyncracy of the patient and the sensitiveness of the part will permit.

6. It is advisable in becoming familiar with its use for the physician to make some experiments upon his own person.

7. All things considered, I think the mothod should be commended for general adoption in minor operations by those in dermatological practice.

